# Mclndoe Neovagina in patients with Mullerian Agenesis: A single center experience

**DOI:** 10.12669/pjms.331.11867

**Published:** 2017

**Authors:** Abdulrahim Gari

**Affiliations:** Dr. Abdulrahim Gari, MD, FRCPC. Assistant Professor, Department Of Obstetrics and Gynaecology, College of Medicine, Umm Al-Qura University, Makkah, and King Faisal Specialist Hospital & Research Centre, Jeddah, Saudi Arabia

**Keywords:** Mullerian aplasia, Mayer-Rokitansky-Kuster-Hauser (MRKH) syndrome, Vaginal agenesis, Vaginal reconstruction, Split thickness skin graft

## Abstract

**Objectives:**

To evaluate the surgical feasibility and the long-term anatomical and functional results and complication rates in patients with Mullerian aplasia who underwent vaginal reconstruction.

**Methods:**

A retrospective observational case series study over 8 years was conducted in King Faisal Specialist Hospital & Research Centre – Jeddah, Saudi Arabia. All cases diagnosed as Mullerian aplasia and who underwent surgical correction were included. Painless and satisfactory vaginal intercourse after surgery were the main outcome measured.

**Results:**

A total of 19 patients were diagnosed with Mullerian agenesis and underwent vaginal reconstruction by Mclndoe technique with minor modification. All of our patients who were sexually active and were compliant with mold use postoperatively were able to achieve 100% painless and satisfactory sexual intercourse.

**Conclusions:**

Minor modification in McIndoe technique provides easier, safer and deeper dissection that minimizes the complications and helps in maximizing the vaginal length. It provides satisfactory and functional vagina in the majority of the patients.

## INTRODUCTION

Mullerian agenesis also named as Mullerian aplasia, Mayer-Rokitansky-Kuster-Hauser (MRKH) syndrome or Vaginal agenesis, is a rare condition with an incidence of 1 in 4,000 to 1 in 10,000 females.^1^,^2^ The general obstetrician and gynecologist can expect to encounter this condition for a few times during their professional career.

It refers to the congenital aplasia or severe hypoplasia of the structures derived from the mullerian duct, including proximal 2/3 of vagina, uterus and fallopian tubes.^3^ In these patients ovaries are normal in structure and endocrine function due to separate embryological origin,^1^ therefore have a normal development of secondary sexual characteristics and external genitalia with normal 46XX karyotype and hormonal profile.^1^,^4^

Mullerian agenesis is the second most common cause of primary amenorrhea in adolescent, after gonadal dysgenesis^1^,^5^ and usually diagnosed at puberty. It is subdivided into two types: Type I isolated form or Rokitansky sequence and Type II or MURCS association (Mullerian duct aplasia, Renal dysplasia, and Cervical Somite anomalies) in which other anomalies coexist.^4^ In type II; more frequently associated anomalies are urological in 15-40% of cases^4^ and skeletal anomalies in 20-40%^4^ while auditory and cardiac defects are infrequent.^4^,^5^

The etiology of Mullerian aplasia still remains unclear. Initially, it was considered as a sporadic anomaly, but the rising number of the familial cases now favors the hypothesis of a genetic etiology where it appears to be transmitted as an autosomal dominant trait with variable expressivity and incomplete penetrance, this suggests either mutations in a major developmental gene or a limited chromosomal imbalance.^4^

The diagnosis of Mullerian aplasia is ready achieved through well-defined diagnostic steps performed for primary amenorrhea at puberty^6^ and has become very efficient due to the ready availability of advanced imaging modalities. However, extensive controversies exist while deciding optimum therapeutic option.

The aim of our study was to evaluate the surgical feasibility and the long-term anatomical and functional results and complication rates in patients with Mullerian aplasia who underwent Mclndoe technique of vaginal reconstruction with minor modification in form of mini-laparotomy which made it easier, safer and allowed a deeper dissection.

## METHODS

A retrospective observational case series study was conducted in King Faisal Specialist Hospital & Research Centre – Jeddah, Saudi Arabia. All cases diagnosed as Mullerian aplasia (Mayer-Rokitansky-Hauser syndrome) and who underwent the surgical correction, i.e., Mclndoe technique of vaginal reconstruction with minor modification (in the form of mini-laparotomy) over 8 years (July 2007 - June 2015) were included.

Patient’s files and electronic medical record system were reviewed. Data was collected by using a preformed Proforma for evaluation of surgical feasibility and anatomical and functional outcome.

Patient’s information regarding preoperative history, clinical examination, abdomino pelvic’s ultrasound, pelvic magnetic resonance imaging (MRI), hormonal profile and chromosomal analysis was collected.

Diagnosis and treatment options were discussed with the patients and their partner/parents. Surgical procedure along with all possible complications, postoperative care (course) and mold usage were explained in detail. Most of the patients were seen by the psychiatrist and counseled about their congenital anomalies and reassured by knowing that they are normal female genotype. After signing informed consent patient were scheduled for surgery. Surgical team includes gynecologist and plastic surgeon (for the skin graft only).

All patients were operated under general and epidural anesthesia (for post-operative pain control) in the lithotomy position with urinary catheterization, the catheter was kept for two weeks postoperatively. Patients were covered with antibiotic, i.e., Ciprofloxacillin and Metronidazole which were started 30 minutes before surgery and continued for two weeks postoperatively. First 48 hours as an IV form then changed to oral form.

The gyne team starts the procedure, after aseptic technique a mini-laparotomy i.e. 5cm transverse incision was made, the peritoneal cavity was entered. Peritoneal fold at the site of missing or non-formed uterus was identified (the diagnosis is confirmed). The assistant continues abdominally in order to direct the surgeon toward the primitive peritoneal fold (the correct direction between the bladder and rectum) and to determine the ideal depth of dissection without entering the peritoneal cavity through the vaginal approach.

The original vagina was identified and incised transversely. A vesicorectal space was created by blunt and occasionally sharp dissection between the bladder and the rectum directed immediately posterior to the peritoneal fold which represents the upper limit of dissection. Meticulous hemostasis of the newly created vagina was secured.

**Fig. 1:**
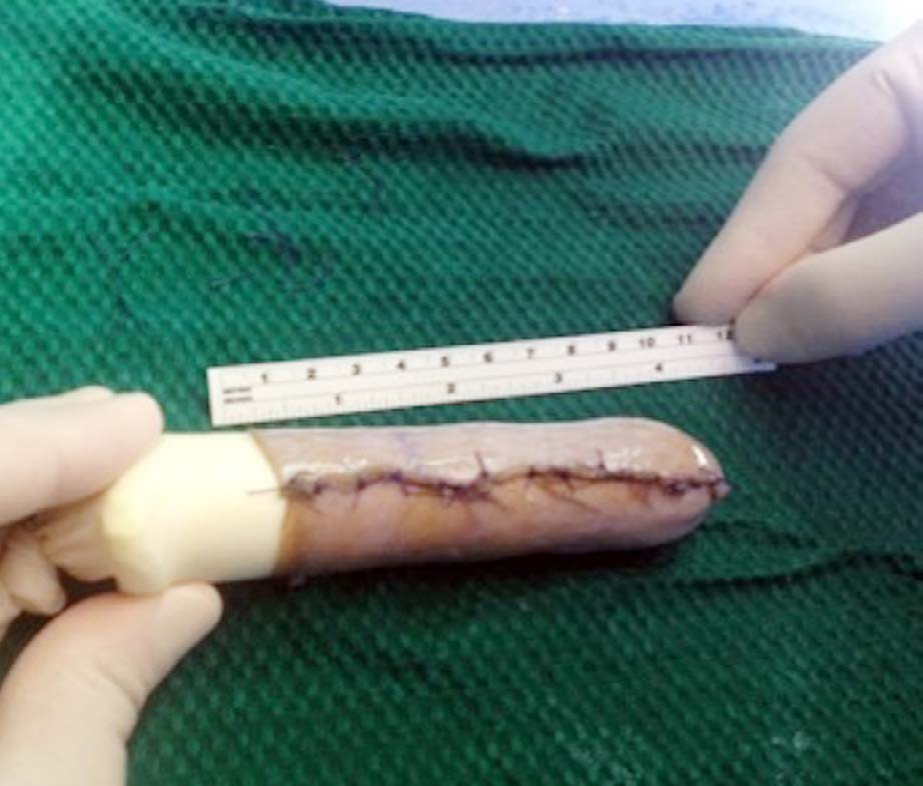
Skin graft on the mold by using absorbable suture 3/0 vicryl.

The plastic surgeon takes over the case and a split thickness skin graft was taken from the lateral aspect of the thigh. The Gyne team continues the procedure and sutures the skin graft on the mold by using absorbable suture 3/0 vicryl (Fig.1). This was then inserted into the prepared neovaginal space and the free edge of the skin graft was sutured to the edge of the perineal incision, forming the neovaginal introits. Finally, the vagina was obliterated for two weeks by permanent sutures (Ethibond sutures). The epidural anesthesia is usually used for three days for pain control then oral analgesics were used.

After two weeks patients were taken back to Operating Theater for examination under anesthesia (EUA) and vaginoscopy. The mold is removed carefully and a Hysteroscope is used for the vaginoscopy part to assess the healing of skin graft. The mold was removed, cleaned and the condom over it was changed. Newly created vagina was irrigated with normal saline and mold was reinserted. Postoperatively after 2^nd^ surgery patients were educated, the points focused on; how to change the condom on the mold, cleaning the mold, it should be done twice a day.

Patients will keep the mold 24 hours a day for up to 6 months. After 6 months when healing is complete, patients were allowed to engage in vaginal intercourse and they can use the mold only at bedtime for the next 6 months. After the first year of procedure, they do not need to use the mold unless there is no vaginal intercourse, in that case, they can use the mold at bedtime.

### Ethical issues

We had taken the permission from the Institutional Review Board for this study.

### Informed consent

All the participants were informed of all potential and future prospects of this survey and their informed consent was taken. All respondents were assured of strict confidentiality of their identity.

## RESULTS

A total of 19 patients were diagnosed with Mullerian agenesis and underwent vaginal reconstruction. Age of these patients at the time of diagnosis varied between 14 to 38 years (mean 21.2 years). Ten patients were married at presentation and eight patients married after surgery. Out of these eight patients who were remarried after surgery, 7 patients were divorced due to reasons related to their diagnosis. One patient (15 years old) remained unmarried. All patients presented with complaint of primary amenorrhea and difficulty in sexual intercourse if they were married or divorced. They have a normal female general appearance, secondary sexual characteristics and external genitalia (female phenotype) however, the lower vagina was felt just as a dimple i.e., 0-2 cm blind pouch. In all the 19 patients there was an absent uterus, cervix, fallopian tubes and upper 2/3ed of the vagina however both ovaries were normal. Four out of 18 patients (21.1%) were diagnosed with renal anomalies. Hormonal profile and chromosomal analysis were normal in all the 19 patients. Pre-operative vaginal length varied from 0-2cm (mean length 0.82 cm).

There was no intraoperative complication i.e., bleeding, bowel, bladder or rectal injury. The peritoneum was never opened during the dissection through the vaginal approach. Operative time ranged from 90 minutes to 240 minutes (mean operative time of 150 min) and the mean estimated blood loss was 150mls. No immediate or late postoperative complication was reported (i.e., haemorrhage, hematoma, urinary tract infection, fistula or keloid scar on donor site). In one patient there was vaginal stenosis after 8 months of procedure, she was divorced and non-compliant with the use of vaginal mold. She was taken back to operative room for examination under anesthesia, dilation and insertion of a mold was easily done. There was a band of fibrosis at the junction between the original and newly created vagina. A small incision was made at (3, 6, 9 and 12 O’ clock position) and sutured, the mold was inserted. After that she did well and was advised to continue to use mold. Postoperative vaginal length varied from 7-17cm (mean length 10.3 cm). Out of 19 patients only two did not engage in regular sexual intercourse. One patient was unmarried while 2nd patient’s husband job was in other city. Patients engaged in sexual intercourse all of them reported satisfactory sexual activity in both partners with no pain and with normal mucosal-like active vagina. The mean follow-up period after surgery was 25 months (range 12 to 60 months).

## DISCUSSION

Mullerian agenesis even if this is rare but reported to be the second most common cause of primary amenorrhea, next only to gonadal dysgenesis.^1^,^5^ Effective management of these patients includes correct diagnosis, evaluation of associated congenital anomalies, and psychosocial counseling before any treatment or surgical intervention. Counseling is important to address the functional and emotional effects of the genital anomalies as well as corrective options available.^1^ Patient’s medical history, preferences, motivation, lifestyle and underlying condition, along with the surgeons’ capability, play an important role in choosing correct approach. However timing for creation of a Neovagina is elective and is best planned when the patient is emotionally mature, highly motivated and express desire for correction. Different non-surgical and surgical techniques are available to treat this anomaly. However, at present there is no consensus in literature regarding the best procedure for surgical correction to afford the best functional outcome and sexual satisfaction.^7^

The Mclndoe procedure is one of the most frequently performed surgical procedure which involves the surgical creation of a space in between the rectum and the bladder, placement of a mold covered with a split-thickness skin graft into the space, and use of vaginal dilators postoperatively. In the past years (last decades) surgical treatment has gradually evolved from aggressive procedures to minimally invasive procedures and different authors have introduced different modifications, mostly changing the lining material with aim to improve the short and long-term results.

We did a minor modification in Mclndoe technique. Using a mini-laparotomy (5cm transverse incision), in order to identify the peritoneal fold at the site of missing or non-formed uterus. This helps the surgeon to know the correct direction and the upper limit of vaginal dissection with less likelihood of opening of peritoneum. This guidance provides easier, safer and deeper dissection hence a longer neovagina with added advantage of fewer complications.

Traction is used instead of dilatation to create neovagina in the Vechietti technique and that is usually performed laparoscopically with potential risk of conversion to laparotomy which is subsequently associated with high complication rate. Furthermore, the traction of the ‘olive’ at vaginal dimple can be painful and may not be easily tolerated by patient.^8^-^10^ Brucker et al.^11^ and Fedel et al.^12^ used Vechietti technique in 101 and 110 patients, respectively but sexual intercourse without dyspareunia was achieved only in 60% of patients. However, by using McIndoe technique 80-90% sexual satisfaction rate was reported^13^,^14^ which is higher than the previously mentioned rate with Vechietti technique.

In our case series all of our patients (17 out of 17) who were sexually active and were compliant with mold use postoperatively were able to achieve 100% painless and satisfactory sexual intercourse. Out of remaining two patients one was unmarried and not engaged in any sexual activity while the other patient had vagina stenosis 8 months after surgery; she was divorced and non-compliant with mold usage. Later after correction of vaginal stenosis she got married and also achieved painless and satisfactory sexual intercourse.

Davydov in 1969 first time used a three staged operation which requires dissection of the rectovesical space with abdominal mobilization of a segment of the peritoneum, and subsequent attachment of the peritoneum to the introitus.^1^ This technique can be performed laparoscopically or by laparotomy. However, there is a significant risk of injury to the bladder, rectum and/or ureter, along with risk of peritonitis and vesicovaginal fistula formation.^15^

Vaginoplasty using intestinal graft is another surgical approach. Although this procedure neither need any postoperative vaginal dilatation nor any risk of postoperative stenosis and had a natural lubricant effect but associated with serious infection, intestinal stenosis, dehiscence, and fistula formation. Furthermore, new vagina formed after intestinal graft will be less sensitive and prone to produce significant mucus which can affect patient social and sexual life as she may need to use sanitary pads continuously. Rare risk of malignancy is another risk needs to remember.^16^-^18^

Other techniques, such as using buccal mucosa, amnion, inert materials and autologous in vitro grow vaginal tissue are less commonly used procedures in patients with Mullarian agenesis. Result of our case series shows that deeper dissection guarantees adequate vaginal length, i.e., more than 8cm that we achieved in all of our patients which allow deeper penetration during sexual activity and help in achieving painless and satisfactory sexual intercourse for both partners. Postoperative compliance with regular and proper mold usage is paramount especially if patients are not sexually active or not engage in frequent or regular sexual activity.

Routine gynecological care is required for patients with Mullerian agenesis and who undergo vaginal reconstruction. Women who are not sexually active should undergo a regular pelvic examination to detect vaginal stricture formation and hence prevention. The women who are sexually active are at a risk of sexually transmitted disease as well and should understand the importance of using condoms and should have appropriate screening according to guidelines for women without Mullerian agenesis. However routine vaginal cytological testing is not recommended but vaginal speculum examination and inspection should be performed to look for any suspicion of malignancies as there is a theoretical risk of vaginal neoplasia and genital warts but at present insufficient evidence is available to guide the decision for human papillomavirus vaccination.^1^

## CONCLUSION

Vaginal reconstruction by Mclndoe technique with minor modification in the form of mini-laparotomy is simple, reliable, safe and effective treatment in patients with Mullerian agenesis. The modification minimizes the complications and helps in maximizing the vaginal length. It provides satisfactory and functional vagina in the majority of the patients. Patients full understanding, motivation and compliance with proper mold usage after surgery remain the mainstay of treatment to get optimal results.
